# Symptom Burden and Intervention Outcomes in Idiopathic Normal Pressure Hydrocephalus: A Multicenter Retrospective Study

**DOI:** 10.7759/cureus.88328

**Published:** 2025-07-19

**Authors:** Joshua Amaya, Zachary D Griffin, Curran Reddy, Jeremy Doan, Grace Wang, Sameer Allahabadi, Daniel Habenicht, John Hunton, Amal Khan, Subhash Venigalla, Daniel Eickenhorst, Shovendra Gautam

**Affiliations:** 1 Graduate Medical Education, Baylor Scott & White All Saints Medical Center, Fort Worth, USA; 2 Burnett School of Medicine, Texas Christian University, Fort Worth, USA

**Keywords:** gender disparity, lumbar puncture (lp), normal pressure hydrocephalus, reversible dementia, ventriculoperitoneal shunt

## Abstract

Introduction: Normal pressure hydrocephalus (NPH) is a potentially reversible cause of dementia, characterized by a clinical triad of gait disturbance, urinary incontinence, and cognitive decline. Despite suggestive radiologic findings, many patients remain untreated. This study aimed to determine the clinical correlation between radiographic findings of NPH and clinical symptoms. It also investigated whether the number of NPH symptoms predicts the likelihood of intervention and clinical improvement (in any of the triad of symptoms), and assessed gender-based disparities in treatment.

Methods: We conducted a multicenter retrospective chart review across three hospitals, identifying patients with radiologic evidence of NPH between 2019 and 2023. We recorded symptom burden, intervention rates (shunt placement or lumbar puncture), and post-intervention outcomes.

Results: Among 385 included patients, both intervention rates and response to therapy increased with symptom count. Gender differences were noted, with men more likely to receive treatment. However, improvement rates did not significantly differ based on the number of symptoms.

Conclusion: Of all patients with radiology findings of normal pressure hydrocephalus, 317 (82.3%) had at least one symptom classically associated with NPH. A proportion of iNPH patients may be undertreated. Symptom burden and gender disparities appear to influence clinical decision-making and warrant further investigation.

## Introduction

Normal pressure hydrocephalus (NPH) is a chronic neurological disorder characterized by an abnormal accumulation of cerebrospinal fluid (CSF) within the cerebral ventricles, leading to their enlargement and subsequent compression of surrounding brain tissue. NPH is divided into two categories: secondary NPH, which is due to identifiable causes like hemorrhage or infection, and idiopathic NPH (iNPH), which excludes such secondary etiologies. iNPH is present in roughly 6% of individuals over the age of 80 [1]. Diagnosis of NPH requires enlarged ventricles and at least a portion of the classical clinical triad of gait disturbance, dementia and urinary incontinence. International guidelines specify additional criteria such as age over 60 years old, Evans’ Index greater than 0.3, clinical triad presence, exclusion of alternative causes of dementia, normal CSF opening pressure, and disproportionate enlargement of the subarachnoid space hydrocephalus (DESH) [2]. Gait disturbance is usually the first symptom, followed by dementia and then urinary incontinence [3]. Importantly, few conditions in the elderly population are associated with such responsiveness to therapy [4]. This is particularly true with dementia symptoms, and NPH has been referred to as reversible dementia [5]. Therefore, our ability to accurately identify and successfully treat NPH is quite important.

Importantly, NPH diagnosis represents a diagnostic challenge for many clinicians as there is heavy clinical overlap with other dementia diseases. Selecting which patients will respond well to intervention is important. In 2012, an international consensus defined diagnostic criteria for iNPH, emphasizing the presence of the clinical triad, ventriculomegaly (Evans Index ≥ 0.3), and improvement with CSF removal [6]. These criteria serve as the basis for current international and Japanese guidelines and form the framework for identifying patients likely to benefit from intervention. While intervention is effective, leading to sustained five-plus-year clinical remission in the majority of patients, it also has significant side effects [7-9]. Many research studies have been dedicated to investigating the prognostic effect of various radiologic findings on outcomes with ventriculoperitoneal (VP) shunts [1,10,11]. However, few have investigated whether the effect of the number of NPH symptoms in patients with any radiologic findings of NPH correlates with intervention rates and therapeutic response.

Therefore, this study aims to characterize the prevalence of classic NPH symptoms (dementia, urinary incontinence, and gait disturbances) in patients initially identified by radiographic findings suggestive of NPH. Building on this, we will then investigate how the number of these symptoms correlates with both intervention rates and subsequent therapeutic response. Furthermore, we will analyze gender-based differences in intervention rates and overall patient outcomes within this cohort.

## Materials and methods

This study is a multi-center, retrospective, cross-sectional study with an aim to correlate the MRI and CT findings in patients with NPH with symptoms of dementia, urinary incontinence and gait disturbances. Radiology reports from three Baylor Scott & White hospitals (All Saints, Irving, and Grapevine) between 2019 and 2023 that included the phrase 'Normal Pressure Hydrocephalus' in the dictation section were identified. This phrase was considered indicative of radiologic findings suggestive of NPH by the interpreting neuroradiologist, often based on features such as Evans Index, presence of DESH, or other signs consistent with NPH. This resulted in 788 scans being identified from a total of 703 patients (some patients had multiple imaging scans performed). These 703 patients had NPH on the differential given imaging findings.

**Figure 1:**
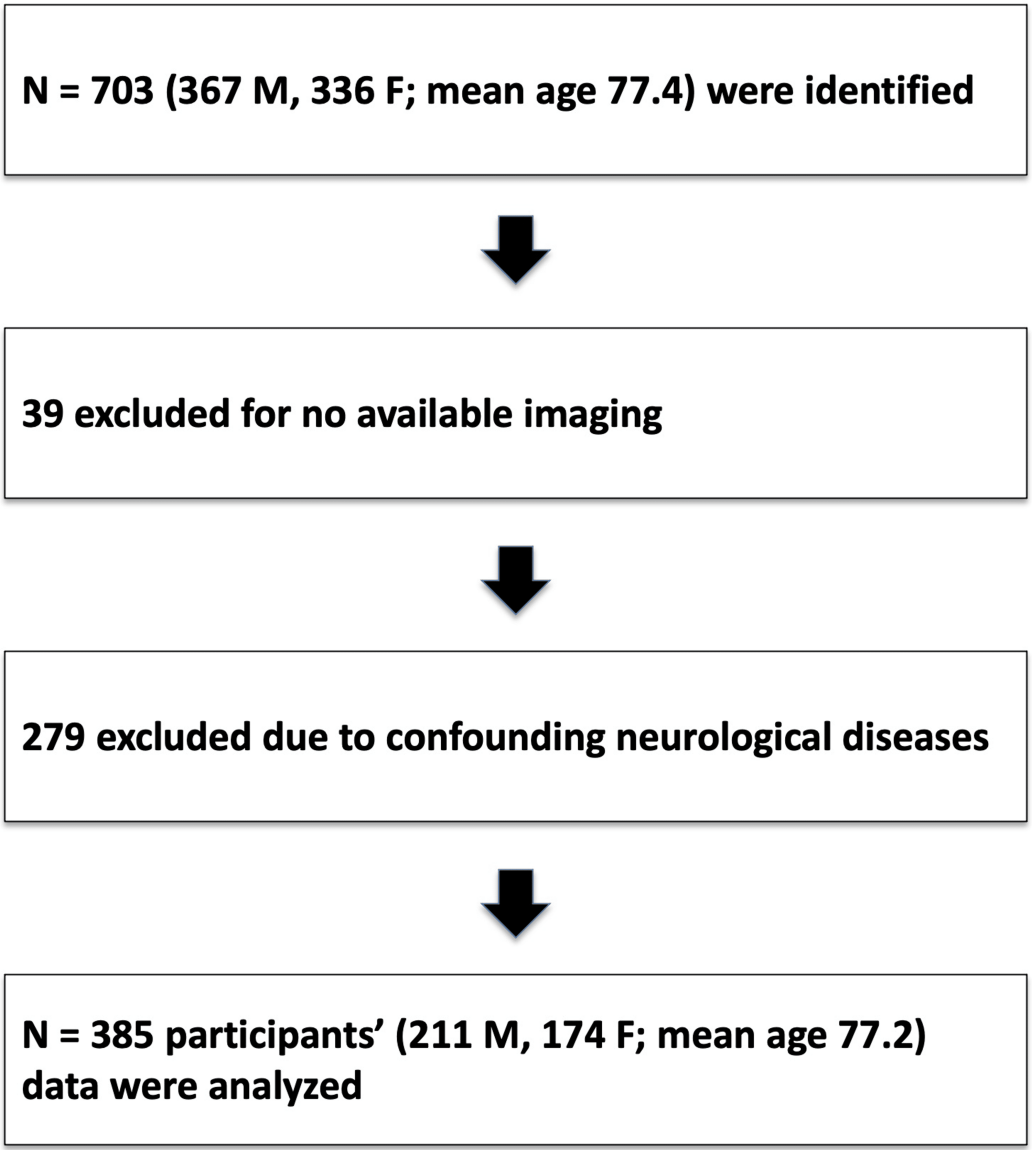
Flowchart of inclusion and exclusion criteria for patients whose data were analyzed in this study

An electronic chart review was conducted for all patients who underwent CT or MRI Head using a standardized radiographic report analysis protocol from September 12, 2019, to August 30, 2023. Patients with no images available and with co-morbid neurologic disease (previous dementia diagnosis, stroke, traumatic brain injury, primary tumor, metastasis) were excluded; however, patients with age-related cerebral atrophy were not excluded. All included patients were selected if they had these initial radiographic findings of NPH and complete data on symptoms and intervention status. Patients with non-neurologic comorbidities such as diabetes and hypertension were not excluded. This left us with 385 remaining patients for analysis. A flowchart with inclusion and exclusion criteria is shown in Figure 1. As this was a retrospective review, all eligible patients meeting inclusion criteria were included. All included patients had complete symptom and intervention data; missing imaging data was grounds for exclusion.

For these 385 remaining patients, an electronic chart review was performed. The prevalence of each of the three classic NPH symptoms (dementia, urinary incontinence, and gait disturbances) was determined by comprehensive review of physician notes, nursing assessments, and available clinical documentation within the electronic chart. While standardized scales were not uniformly available due to the retrospective nature of the study, symptom ascertainment relied on the independent clinical judgment of the reviewing physicians based on documented evidence. Additionally, patient demographics (gender), interventions performed, and post-intervention outcomes were recorded. For a small number of these patients (20), the Evans Index was identified and measured by three different physician readers, under the supervision of a board-certified radiologist. Intraclass Correlation Coefficient (ICC) was calculated for the Evans Index findings.

Clinical improvement was determined based on chart review by independent physicians. Clinical improvement was operationally defined as documented improvement in at least one of the three classic NPH symptom domains (dementia, urinary incontinence, or gait disturbance). Post-intervention assessment of improvement primarily utilized results from standardized testing (e.g., gait testing associated with lumbar puncture) when available. However, due to the retrospective nature of the study, the specific methodology for assessing improvement was variable across patients and involved the independent clinical judgment of the reviewing physicians based on overall documented change.

Statistical analysis included the Two-Proportion Z-Test to compare intervention rates between males and females, and Fisher’s Exact Test to evaluate the relationship between symptom count and intervention effectiveness. Fisher’s Exact Test was chosen due to the small sample size and low expected counts in some groups, making a Chi-Square Test unsuitable. A post hoc power analysis was performed to assess the study’s ability to detect differences in intervention effectiveness across symptom groups. Given the retrospective nature of the study, no prospective power calculation was done. Improvement rate confidence intervals were also calculated using Wilson score intervals to provide better estimates of precision, particularly for smaller subgroups.

Institutional Review Board (IRB) approval (393930) was granted by Baylor Scott & White’s Research Institute IRB via expedited review in October 2023. It was categorized as Category 5 research and the IRB waived the requirement for informed consent based on 45 CFR 46.116 (f).

## Results

In total, 788 radiographic scans matched our search criteria (547 CT scans, 231 MRI). Seven hundred three patients were identified. Of these patients, 39 participants were excluded as no CT or MRI imaging was available in the chart. Two hundred seventy-nine participants were excluded due to confounding neurological diseases. ​

**Figure 2:**
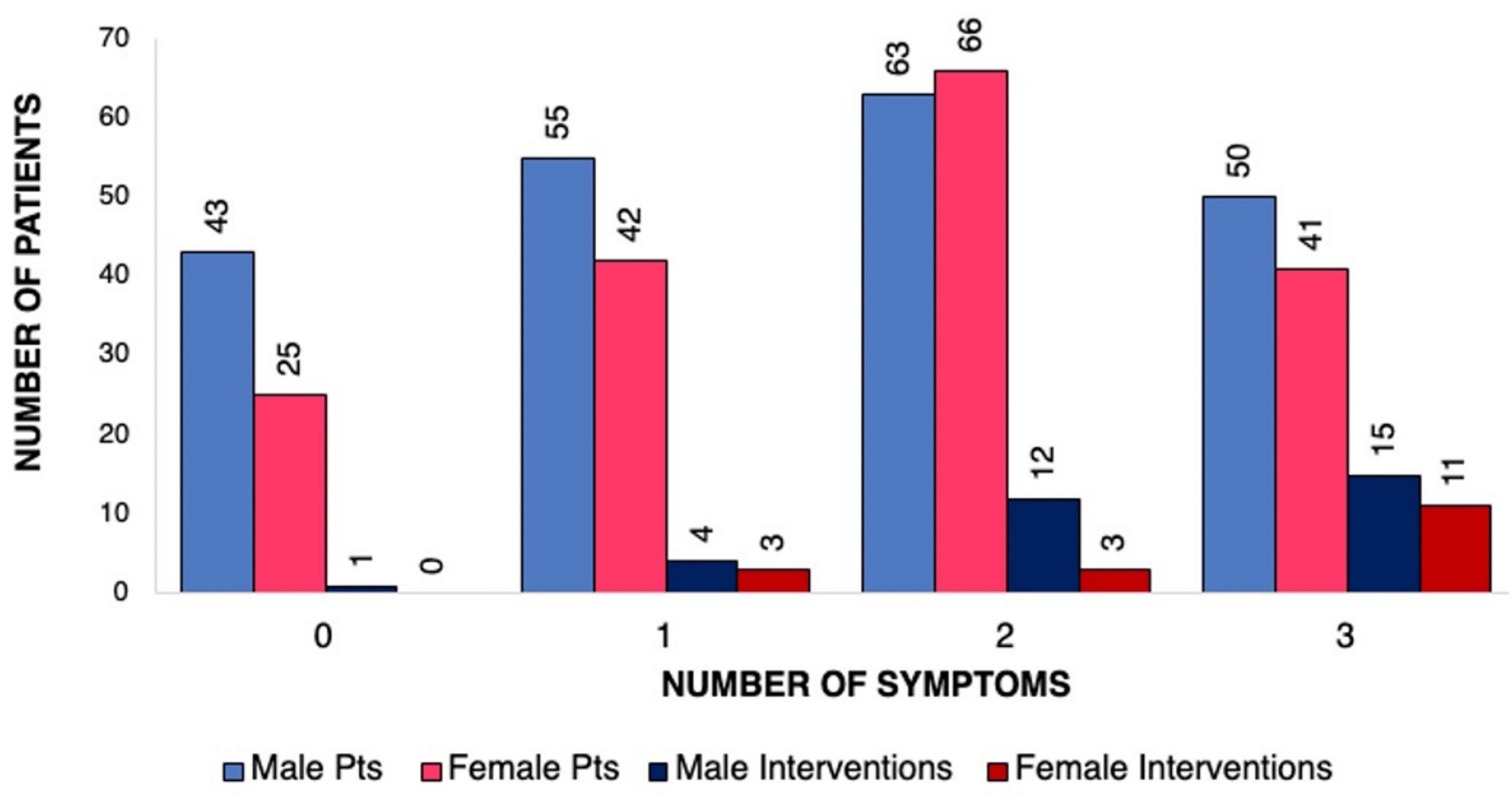
Symptom Burden and Intervention Rates Among Patients With Radiologic iNPH Findings, Stratified by Sex Bar chart showing the distribution of male and female patients with radiographic findings of idiopathic normal pressure hydrocephalus (iNPH), categorized by the number of presenting clinical symptoms (zero to three). For each symptom count, the first two bars represent the total number of male (light blue) and female (pink) patients. The next two bars show how many patients in each group underwent an intervention (lumbar puncture or shunt), with male interventions in dark blue and female interventions in red. This figure illustrates both symptom burden and sex-based differences in intervention rates across symptom categories.

Overall, imaging results and records from 385 participants (211 M, 174 F; mean age 77.2) were included in our analysis. Of those patients with imaging findings consistent with NPH, 68 (17.7%), 97 (25.2%), 129 (33.5%), and 91 (23.6%) had zero, one, two, or three of the classic NPH symptoms. Three hundred seventeen (82.3%) out of 385 patients with imaging findings consistent with NPH had one or more symptoms. The number of symptomatic patients who underwent intervention is shown in Figure 2. There was a significant difference (p-value of 0.008) in overall intervention rates between males and females (Figure 2). When stratified by number of symptoms, there was no difference in intervention rate between men and women for patients with zero or one symptoms; however, there was in those with two or three symptoms. Of those with three symptoms, six of 11 (55%) females and six of 15 (40%) males showed improvement in at least one symptom following any intervention.

**Figure 3:**
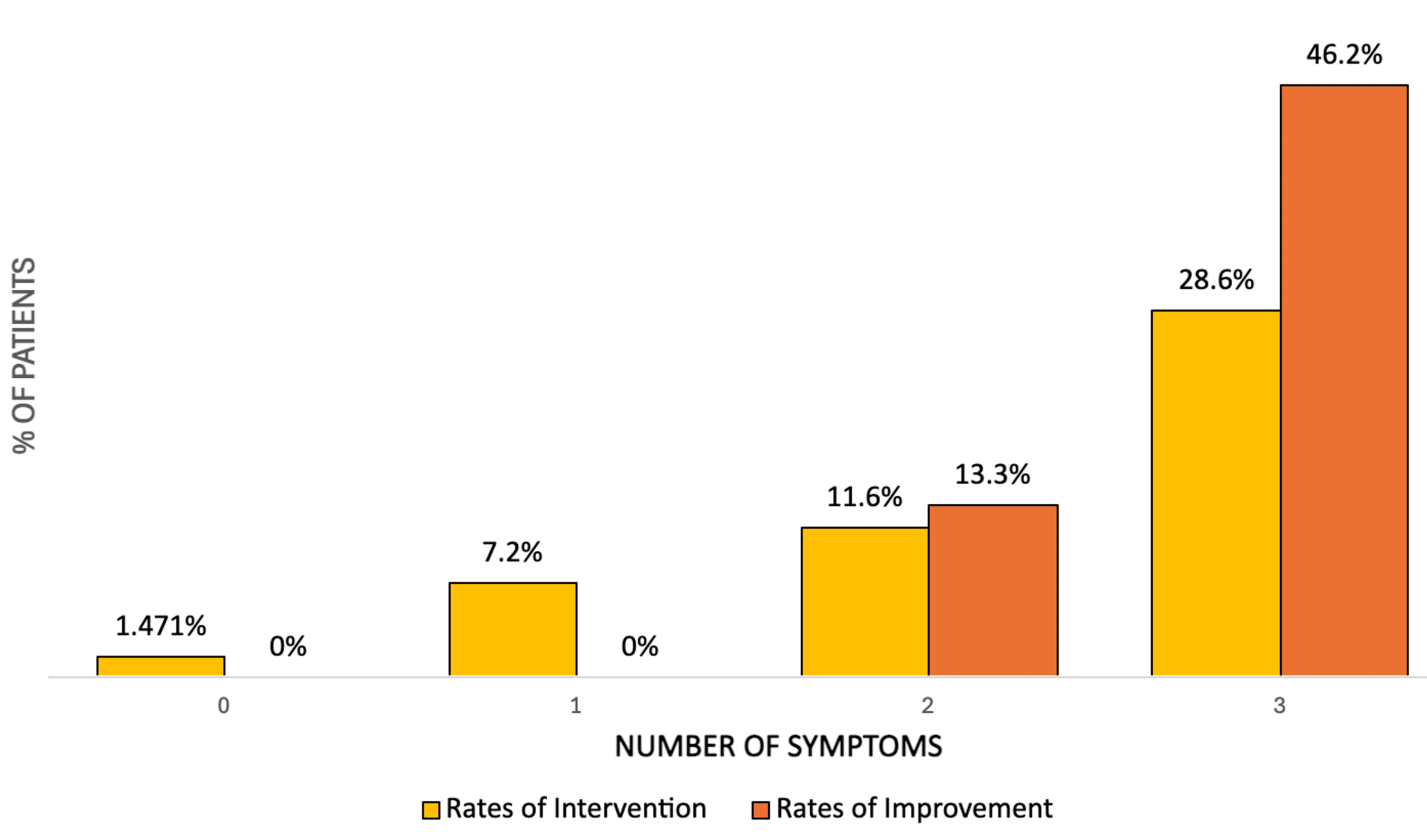
Percentage of patients who underwent intervention and had subsequent improvement stratified by number of clinical triad symptoms. The differences in rate of improvement by number of symptoms are visual differences, not true difference, as the differences did not reach statistical significance.

​​Lumbar puncture or VP shunt/drain was performed in one (1.5%), seven (7.2%), 15 (11.6%), and 26 (28.6%) patients with zero, one, two, or three symptoms, respectively (Figure 3). The percentage of patients with a successful response to intervention increased as the number of symptoms increased. Overall, Fisher’s Exact Test showed no statistically significant difference in intervention effectiveness across symptom groups, with all p-values exceeding 0.05. The odds ratios for intervention effectiveness ranged from 0.00 to 2.31, with corresponding p-values of 0.18, 0.16, and 0.13 for one, two, and three symptoms, respectively. Although patients with more symptoms appeared more likely to undergo intervention and demonstrate some improvement, this trend did not reach statistical significance.

The 95% confidence intervals for the observed improvement rates in one or more symptoms were wide, reflecting the small sample sizes within each symptom group. For the two-symptom group (n = 15), the improvement rate was 13.3% (n=2), with a 95% confidence interval of 3.7% to 37.9%. In the three-symptom group (n = 26), the improvement rate was 46.2% (n=12), with a 95% confidence interval of 28.8% to 64.5%. Post hoc power analysis comparing the two-symptom and three-symptom groups yielded a calculated power of 58.3%.

For a subset of 21 patients included in this study, Evans Index measurements were performed. Of these, 20 patients (95.2%) exhibited an Evans Index greater than 0.3. The inter-rater reliability for Evans Index calculation among the three independent physician readers was quantified with an ICC of 0.97. Additionally, the callosal angle (CA) was measured in 19 of these same patients, yielding an average angle of 75 degrees. Sixteen of these 19 patients (84.2%) had callosal angles of less than 90 degrees.

## Discussion

Of all patients with radiologic findings of normal pressure hydrocephalus, 317 (82.3%) had at least one symptom classically associated with NPH. In the patients with the classical clinical triad of symptoms, 26 (28.6%) underwent intervention with 12 (46.2%) of these patients showing significant improvement in symptoms. There was a clear trend that as the number of interventions increased, the effectiveness of the intervention improved. However, this was not statistically significant in our study. While this trend did not reach statistical significance, the relatively low post hoc power (58% for two- and three-symptom groups) suggests that the study may have been underpowered to detect a true difference. Confidence intervals for improvement rates were also wide, further highlighting the uncertainty in subgroup comparisons. These findings underscore the need for larger studies to more definitively assess the relationship between symptom burden and treatment response.

Multiple large observational studies have noted that men are disproportionately represented among patients receiving intervention for idiopathic normal pressure hydrocephalus, despite no consistent evidence of higher disease prevalence. Population-based MRI studies have shown that NPH affects men and women at similar rates, yet men are more likely to be diagnosed and treated, often comprising 55-60% of surgical cohorts [12-14]. Women present with greater functional impairment by the time of diagnosis, suggesting a delay in recognition or referral, as suggested by data from Sundström et al. [12]. One study suggests that presenting symptoms between men and women may be different, with men presenting with gait disturbance while women present with cognitive disease, which may lead to a difference in work-up [15]. Men often have more comorbidities at time of diagnosis, possibly resulting in greater urgency to treat [12].

Other hypotheses for gender disparity include symptom misattribution (e.g., gait disturbance due to aging or arthritis in women), under-recognition of NPH symptoms in female patients due to under-reporting or clinician bias and additional co-morbidities in men. Importantly, once treated, women demonstrate clinical improvement comparable to men, underscoring the need for greater awareness and timely diagnosis across both sexes [12,14].

These findings align with prior literature showing that although shunting is effective in many cases, its long-term benefit is variable. A 2015 evidence review reported that fewer than half of patients demonstrate sustained improvement in all three classic symptoms at 18 months post-shunt, and emphasized that most supporting studies are observational in nature [16]. Our observed improvement rate of 46.2% (n=12) in patients with the full clinical triad is consistent with this, suggesting that even patients selected for treatment may not uniformly respond. This highlights the need for predictive tools to optimize candidate selection.

The international consensus also recommends CSF drainage testing (e.g., lumbar puncture or external lumbar drainage) as both a diagnostic and prognostic tool [17]. Importantly, studies have demonstrated that proper tap testing, including pre- and post-procedure standardized gait assessment, offers high predictive value for shunt responsiveness [6]. Integration of such assessments may improve the selection process in clinical practice. Future research could explore how consistent application of these guidelines might address the observed under-treatment trends.

Despite the symptomatic nature of the vast majority of patients, an intervention with LP or shunt placement was only performed in a small number of patients (Figure 3). Of the relatively small percentage of patients who underwent intervention, a high percentage showed clinical improvement. This indicates that, at our institution, we are potentially underperforming these interventions. The reasons for this are incompletely assessed by our study. Some possibilities include other diagnoses being higher on the differential due to additional radiographic findings or clinical symptoms not assessed in our study, comorbidities leading to patients being poor candidates for intervention, provider education, or other difficulties in obtaining intervention. However, additional root cause analysis studies at our facility appear to be indicated based on the findings in our study.

There are a variety of imaging findings indicative of normal pressure hydrocephalus imaging findings including Evans Index greater than 0.3, CA, anterior callosal angle (ACA), and anteroposterior diameter of the lateral ventricle index (ALVI) [18]. The imaging findings of NPH are often nonspecific. One study found that CA is the most important imaging finding in distinguishing NPH from mimics of disease [19]. Multiple studies have investigated which of the radiologic findings has the best prognostic value. Imaging findings of an Evans Index higher than 0.3, more acute CA, ACA, wide temporal horns, and DESH signs and score were all associated with better shunt surgery outcome [11,18,20-24]. In our study, only a few of the cases were investigated to identify the specific radiologic findings that led the radiologist to identify NPH in the differential. Of those cases, Evans Index higher than 0.3 was seen in all but one case. The average CA was 75 degrees. Future research should continue to investigate the prognostic implications of the various radiologic findings.

While our study was performed at multiple hospitals within the same healthcare system, many of our findings are likely generalizable to a wider nationwide audience. Clinicians, particularly those treating elderly patients with NPH symptoms, should consider whether they are under-treating NPH in their practice given relatively high response rate to intervention. Patients noted to have NPH in their radiology reports should consider discussing with their physician whether a trial of NPH intervention is warranted in their scenario. Additionally, it would be prudent to consider a quality improvement study at our institution to see if more aggressive NPH interventions would lead to improved clinical outcomes.

Limitations of our study include it being performed exclusively at Baylor Scott and White hospitals; however, this study included patients from multiple hospital sites over a four-year period, increasing the generalizability of our findings. Additionally, the retrospective nature of this study limits its conclusions as it was not a randomized-controlled study; however, this design allowed for the inclusion of a large number of patients with real-world clinical data. The study also relied on electronic medical records for symptom documentation, which may introduce bias due to incomplete documentation or clinician-dependent symptom recognition. Finally, while the study had a relatively small sample size for some subgroups, it still provided valuable insights into intervention patterns and symptom prevalence in a real-world setting.

## Conclusions

NPH is a prevalent and potentially reversible disease, making the identification of suitable candidates for intervention critically important. Our findings reveal that 82.3% of patients with radiographic NPH indications are symptomatic. A significantly higher percentage of men underwent intervention as compared to women. Interventions benefited approximately half of symptomatic patients, particularly those with the full clinical triad. Potentially, given the high response rate, more could benefit from NPH intervention. As such, there is a need for improved understanding of prognostic factors for NPH intervention, improved diagnostic pathways and confirmatory prospective studies.
